# Amphiregulin activates human hepatic stellate cells and is upregulated in non alcoholic steatohepatitis

**DOI:** 10.1038/srep08812

**Published:** 2015-03-06

**Authors:** Chad McKee, Barbara Sigala, Junpei Soeda, Angelina Mouralidarane, Maelle Morgan, Gianluigi Mazzoccoli, Francesca Rappa, Francesco Cappello, Daniela Cabibi, Valerio Pazienza, Claire Selden, Tania Roskams, Manlio Vinciguerra, Jude A. Oben

**Affiliations:** 1 Institute for Liver and Digestive Health, University College London, Royal Free Hospital, London, UK; 2 Department of Medical Sciences, IRCCS “Casa Sollievo della Sofferenza”, S. Giovanni Rotondo (FG), Italy; 3 Dipartimento di Scienze Giuridiche della Società e dello Sport, Università degli Studi di Palermo, Palermo, Italy; 4 Dipartimento di Biomedicina Sperimentale e Neuroscienze Cliniche, Università degli Studi di Palermo, Palermo, Italy; 5 Istituto Euro-Mediterraneo di Scienza e Tecnologia (IEMEST), Palermo, Italy; 6 Dipartimento di Patologia, University of Palermo, Italy; 7 Gastroenterology Unit, IRCCS “Casa Sollievo della Sofferenza”, S. Giovanni Rotondo (FG), Italy; 8 Department of Pathology, Laboratory of Morphology and Molecular Pathology, University Hospitals of Leuven, Leuven, Belgium

## Abstract

Amphiregulin (AR) involvement in liver fibrogenesis and hepatic stellate cells (HSC) regulation is under study. Non-alcoholic fatty liver disease (NAFLD) and its more severe form non-alcoholic steatohepatitis (NASH) may progress to cirrhosis and hepatocellular cancer (HCC). Our aim was to investigate *ex vivo* the effect of AR on human primary HSC (hHSC) and verify *in vivo* the relevance of AR in NAFLD fibrogenesis. hHSC isolated from healthy liver segments were analyzed for expression of AR and its activator, TNF-α converting enzyme (TACE). AR induction of hHSC proliferation and matrix production was estimated in the presence of antagonists. AR involvement in fibrogenesis was also assessed in a mouse model of NASH and in humans with NASH. hHSC time dependently expressed AR and TACE. AR increased hHSC proliferation through several mitogenic signaling pathways such as EGFR, PI3K and p38. AR also induced marked upregulation of hHSC fibrogenic markers and reduced hHSC death. AR expression was enhanced in the HSC of a murine model of NASH and of severe human NASH. In conclusion, AR induces hHSC fibrogenic activity via multiple mitogenic signaling pathways, and is upregulated in murine and human NASH, suggesting that AR antagonists may be clinically useful anti-fibrotics in NAFLD.

Non-alcoholic fatty liver disease (NAFLD), the commonest cause of chronic liver disease in affluent countries, is a spectrum of liver diseases ranging from hepatic steatosis (simple intrahepatic accumulation of lipid droplets) through steatosis with inflammation and fibrosis (non-alcoholic steatohepatitis, NASH) to cirrhosis and hepatocellular cancer (HCC)^1^,^2^. Hepatic stellate cells (HSC), found in the space of Disse (perisinusoidal space between sinusoids and hepatocytes), are the predominant fibrogenic cells in the liver, are activated by liver injury to trans-differentiate from a quiescent state to proliferative matrix producing myofibroblasts^3^. Excessive matrix production may result in cirrhosis with the possibility of HCC onset, although HCC may also develop in a minority of cases in a background of NAFLD/NASH without cirrhosis^4^,^5^.

The epidermal growth factor receptor (EGFR) ligand amphiregulin (AR) plays a central role in branching morphogenesis in organs and is expressed both in healthy and in cancer tissues. It is an autocrine growth factor as well as a mitogen for fibroblasts and regulatory T-cells^6^. Various studies have highlighted the functional role of AR in multiple aspects of tumorigenesis, including transducing growth signals, modulating tissue invasion and metastasis, angiogenesis, and resistance to apoptosis^7^. AR participates in the modulation of the hepatic acute-phase reaction that occurs during inflammation and liver regeneration, and is important for allowing normal hepatocellular proliferation and the restoration of homeostasis^8^. AR has also been shown to be a trigger of liver regeneration after partial hepatectomy^9^,^10^.

The association of AR expression with liver disease has been demonstrated in different animal models of liver damage and in human samples. AR is induced in the fibrotic liver of mice chronically treated with CCl4 and in models of acute liver damage induced by CCl4, activation of Fas and LPS administration, AR induction was also demonstrated in the liver of cirrhotic patients and rats, as well as in human HCC, suggesting that AR is also implicated in hepatic carcinogenesis^9^,^11^,^12^,^13^,^14^. Moreover, it has been suggested to protect from immune mediated liver injury^10^. Interestingly, AR has been suggested to be pro-fibrogenic since mice lacking AR develop less hepatic fibrosis after carbon tetrachloride challenge compared to wild type littermates^15^. However, our understanding of AR function in NASH is far from being complete and, importantly, whether and how AR regulates human (h) HSCs, the major cellular determinant of hepatic fibrosis in NASH, and plays a role in demographically important specific human liver diseases such as NAFLD/NASH is under study.

In this study, our aims were primarily to study the *ex vivo* role of AR signaling mechanisms in the fibrogenic physiology of primary hHSC, and secondarily to determine the *in vivo* significance and disease relevance of these findings by assaying AR in the HSC compartment of a mouse model of NASH and of human livers with NAFLD/NASH at different stages of severity. We show for the first time that AR is expressed in hHSC; further and importantly, we describe AR up-regulation in the HSC of mice and patients with NASH.

## Results

### AR expression in hHSC increases in early passages and parallels ASMA expression and TACE activation

**Figure 1:**
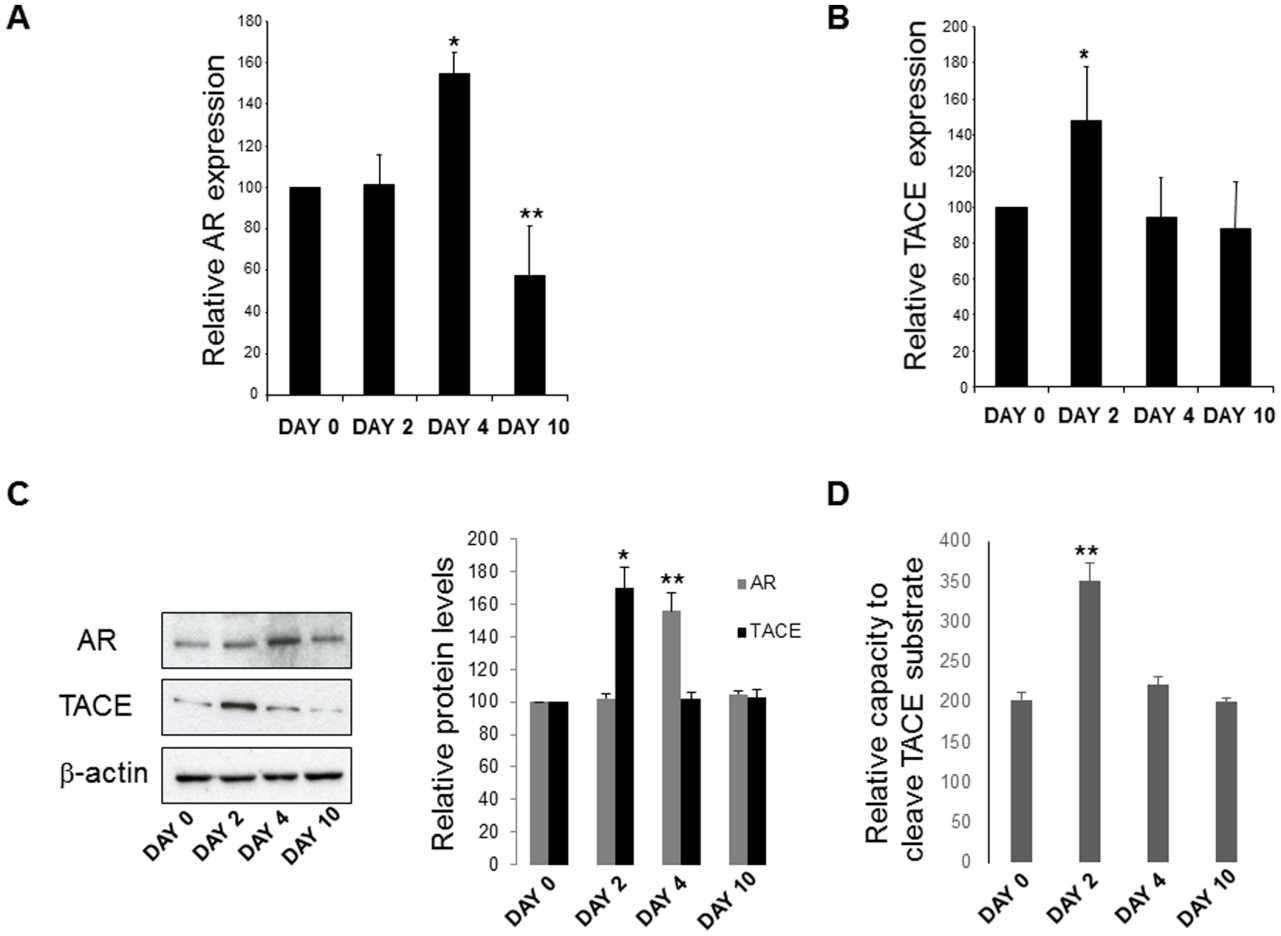
AR mRNA expression in hHSC. (A) Cultures of hHSC show significantly higher expression (* p < 0.05) of AR by day 4 compared to day 0. Expression is effectively reduced by day 10 (** p < 0.01). (B) AR mRNA expression is preceded by the up-regulation of the TACE gene, required for AR activity. TACE expression is highest at day 2 (*p < 0.05) and is reduced by day 4 (**p < 0.01). (C) Western blot analysis for AR and TACE protein levels in hHSC at day 0, 2, 4 and 10 (left panel); images were obtained from separate gels, run under the same experimental conditions, blotted on separate PVDF membranes and processed for detection with the respective antibodies. Densitometric quantification normalized to β-actin levels (right panel). Images are representative of three independent experiments (* p < 0.05; ** p < 0.01). (D) TACE activities in hHSC at day 0, 2, 4 and 10 were measured using SensoLyte 520 TACE Activity Assay kit. N = 3, ** p < 0.01.

**Figure 2:**
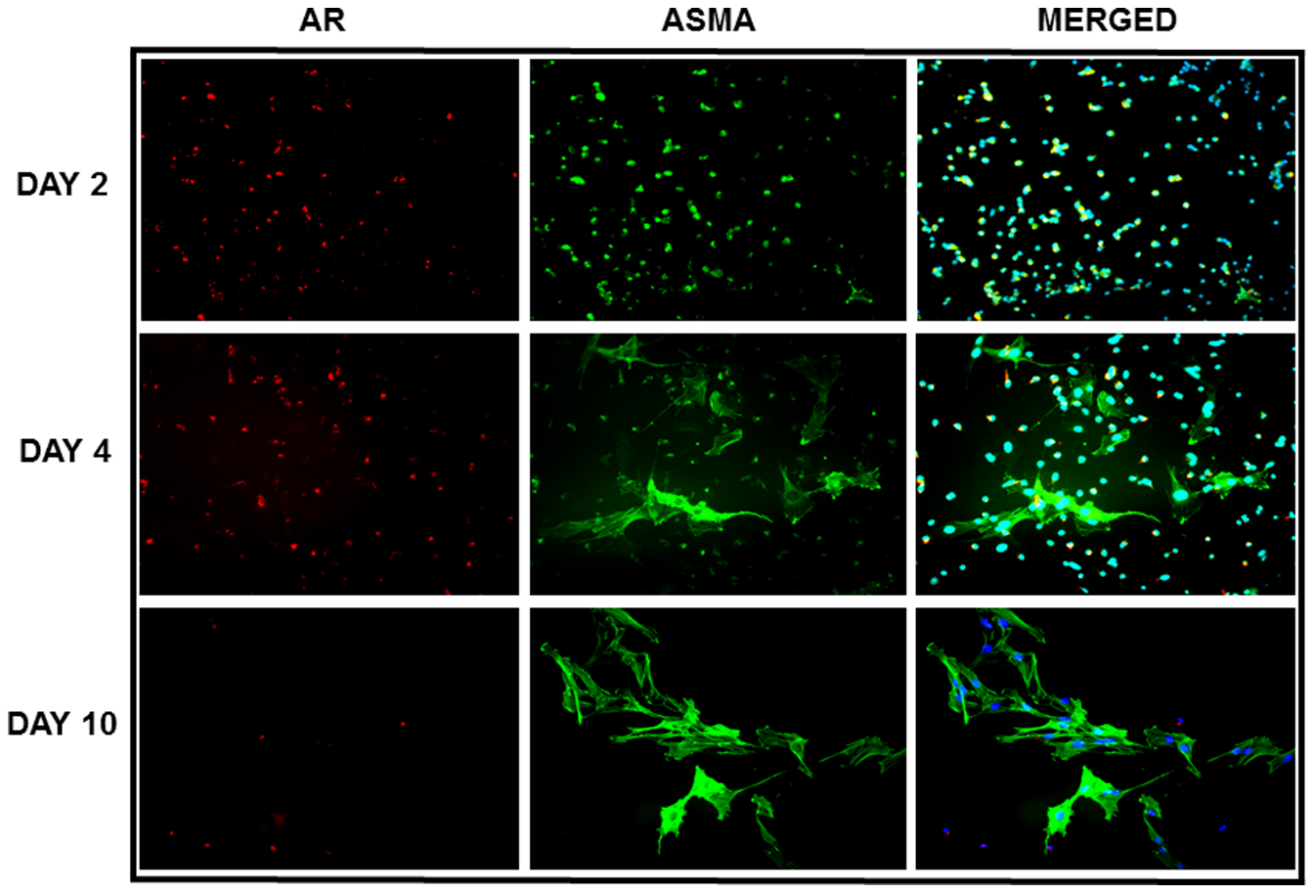
AR expression in hHSC detected by immunofluorescence. Myofibroblastic transformation in culture at day 2 and 4 resulted in a transient increase in AR complemented by α-smooth muscle actin (ASMA) expression, a known indicator of fibrosis. AR was reduced by day 10 in culture while ASMA remains.

AR has been detected in the whole liver but has not previously been reported specifically in HSC. To determine if AR is expressed by freshly isolated (quiescent) HSC and activated HSC we analysed hHSC for AR mRNA and protein expression, as shown in Figure 1: on days 0 and 2 when quiescent, and day 4 and 10 when activated. hHSC expressed AR mRNA and protein, with expression levels at day 4 greater than that at day 10, at which time AR expression became lower at the mRNA level and returned to basal as for protein levels (Figure 1A and 1C). We then analysed the mRNA and protein expression of the AR regulating factor tumour necrosis factor (TNF) α converting enzyme (TACE), which is known to solubilise AR and promote its interaction with its cognate receptors, including EGFR^16^. We found that TACE was expressed by hHSC and its mRNA, protein and activity levels reach all peak values preceding the one of AR mRNA and protein (Figure 1B, 1C and 1D), suggesting that in hHSC TACE is likely to be required for activation of AR. These observations were corroborated by immunocytochemistry data showing that AR expression was confirmed at peak levels at day 2–4, with expression at day 10 lower than that at day 4 (Figure 2). Parallel analysis of the expression of α-smooth muscle actin (ASMA), a marker of HSC activation^17^, showed a progressive increase from day 0, clearly indicating that AR expression preceded that of ASMA which increased progressively from day 0, indicating that AR expression preceded that of ASMA (Figure 2). Our findings also clearly show that hHSC were AR producing cells because of the observed co-localisation of ASMA and AR (Figure 2).

### Exogenous AR stimulates proliferation of hHSC

**Figure 3:**
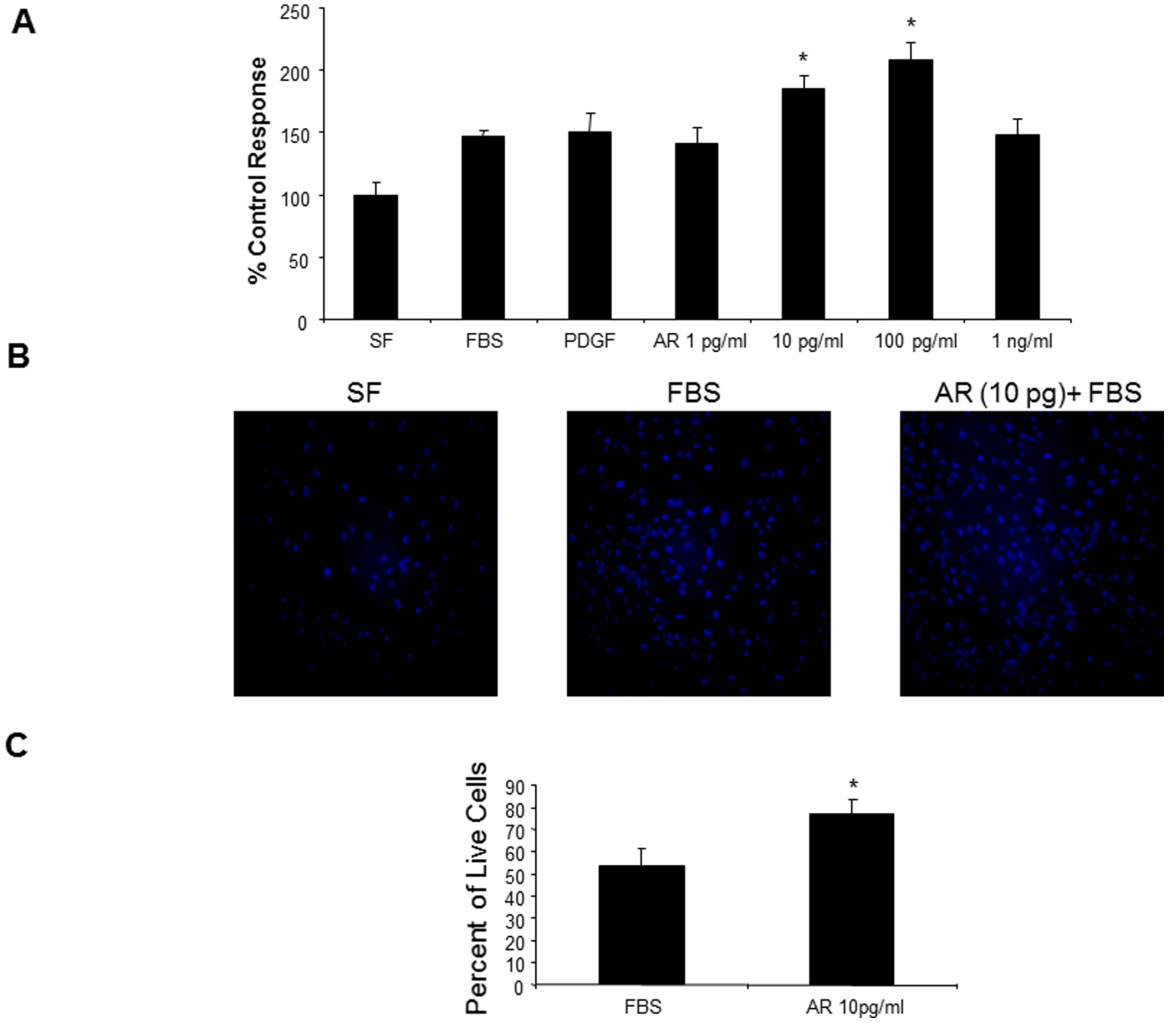
Effect of exogenously added AR on hHSC proliferation. (A) Primary hHSC treated with AR in the picogram range (10 & 100 pgs) are induced to proliferate. Cell division is significantly increased (* p < 0.05) compared to FBS (10%) control and to a known established stellate cell mitogen, platelet derived growth factor (PDGF, 20 ng/ml). (B) Results were confirmed by DAPI staining which enumerated the number of new nuclei in FBS-treated cells or AR (10 pg) + FBS − treated cells. (C) AR treatment (10 pg) of primary hHSC resulted in higher live cell populations as determined by flow cytometry.

AR has been suggested to be pro-fibrogenic because mice lacking AR develop less hepatic fibrosis after carbon tetrachloride challenge compared to controls^15^, but whether this effect of AR is directly dependent on HSC is not known. Here, we found that AR significantly enhanced hHSC proliferation with a maximal effect at 100 pg/ml (Figure 3A). To further explore these proliferation effects, equal numbers of hHSC were seeded into 96-well plates and grown under serum free (SF) conditions, 10% fetal bovine serum (FBS) or with AR (10 pg/mL) plus 10% FBS. As expected, an increased number of cell nuclei was visible between FBS and FBS plus AR (10 pg/mL) conditions compared to SF (Figure 3B). In addition, annexin-PI staining by flow cytometry demonstrated a significantly higher number of live cells in the AR + FBS condition compared to FBS alone (Figure 3C).

### AR-induced hHSC proliferation requires EGFR and the PI3K, MEK, p38 MAPK, PKC survival pathways

**Figure 4:**
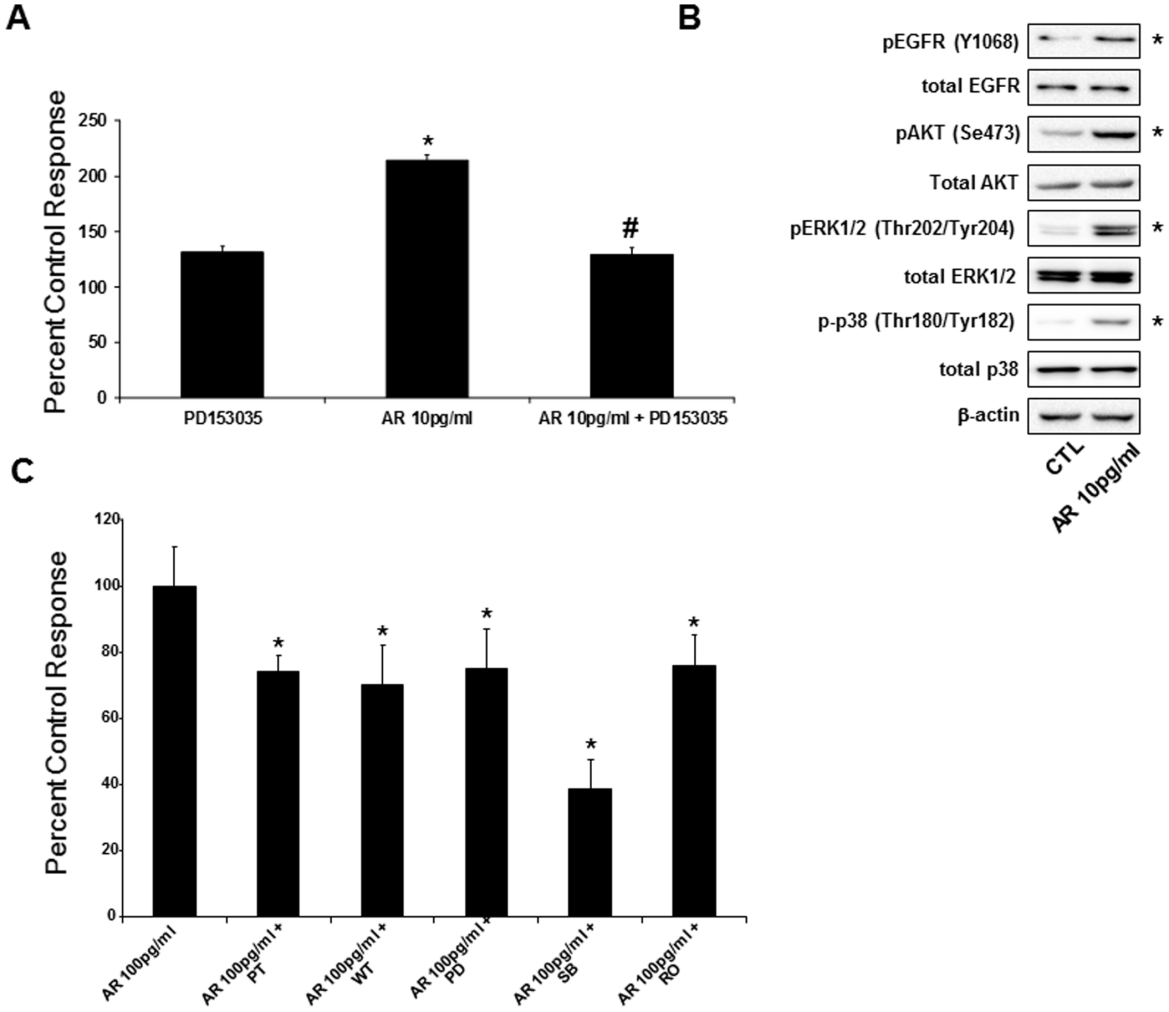
Inhibition of AR-induced proliferative pathways in hHSC. (A) Proliferation was assessed via ELISA with and without EGFR receptor blocker PD153035 (1 μmol/L). AR induced hHSC proliferation (*p < 0.001) that was prevented by blocking the EGF receptor (# p < 0.05). (B) Western blot analysis for pEGFR(Y1068), EGFR, pAKT(Ser473), AKT, pERK1/2(Thr202/Tyr204), ERK1/2, p-p38(Thr180/Tyr182), p38 and β-actin was assayed in primary hHSC cultured with AR (10 pg/ml). Images are representative of three independent experiments. Images were obtained from separate gels, run under the same experimental conditions, blotted on separate PVDF membranes and processed for detection with the respective antibodies. Asterisks indicate increased phosphorylation levels in presence of AR. (C) Involvement of mitogenic intracellular pathways in AR-induced hHSC proliferation was assayed in primary hHSC cultured with AR and in the presence of specific inhibitors: pertussis toxin (G-protein inhibitor, 100 nM), wortmannin (PI3K inhibitor, 100 nM), PD98059 (MEK inhibitor, 100 nM), SB202190 (p38 MAPK inhibitor, 10 μM), and Ro-32-0432 (PKC inhibitor, 1 μM) (*p < 0.001).

AR is thought to act through the EGFR in non-liver cells^18^, but whether its action in hHSC is similarly mediated via EGFR is not known. We show here that treatment with EGFR antagonist PD153035 markedly inhibits AR-induced hHSC proliferation (Figure 4A), pointing for the first time to a role for EGFR in AR-induced hHSC proliferation. Previous studies have shown that AR influenced the phosphorylation of one or more growth signals and MAP kinases involved in the proliferation of vascular smooth muscle cells^19^. Consistently, here we find that culturing hHSC with AR (10 pg/mL) triggers activation of EGFR, AKT, ERK1/2 and p38 MAP kinases, as evidenced by increased phosphorylation levels on key residues [pEGFR(Y1068), pAKT(Ser473), pERK1/2(Thr202/Tyr204) and p-p38(Thr180/Tyr182)] (Figure 4B).To confirm that several mitogenic pathways, other than EGF signalling, are involved in AR-induced hHSC proliferation, we cultured hHSC with AR in the presence of various specific inhibitors^20^: pertussis toxin (G-protein inhibitor, 100 nM), wortmannin (PI3K inhibitor, 100 nM), PD98059 (MEK inhibitor, 100 nM), SB202190 (p38 MAPK inhibitor, 10 μM), and Ro-32-0432 (PKC inhibitor, 1 μM). Our data show that blockage of intra-cellular mitogenic kinases invariably dampen AR-induced hHSC proliferation (Figure 4C). The most potent reductions were observed after treatment with wortmannin and SB202190, indicating that the PI3K and p38 MAPK pathways are of particular importance in AR-induced hHSC proliferation (Figure 4C).

### AR induces fibrogenic markers in hHSC and is upregulated in a murine model of NASH

**Figure 5:**
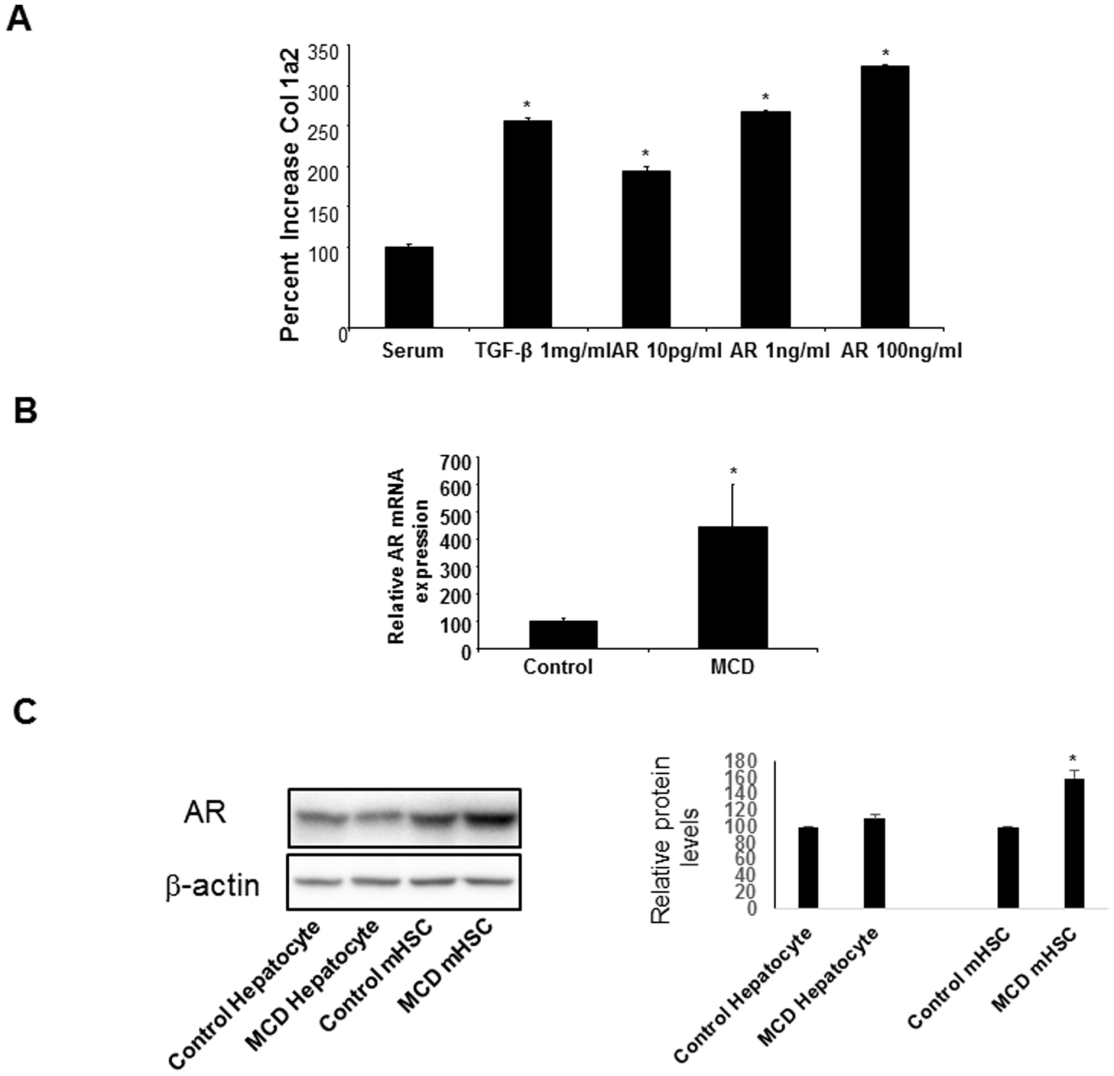
Effect of AR on collagen 1a2 production. (A) AR is capable of inducing collagen production at both picogram and nanogram concentrations (*p < 0.05). The effect is similar to that of TGF-β, inductor of collagen production, a major step in the pathogenesis of fibrosis. (B) Mice were fed either a normal chow diet or a methionine-choline deficient (MCD) diet modelling NASH. Mice on MCD diet expressed higher hepatic AR mRNA levels than mice fed a normal chow diet (*p < 0.001). (C) Western blot analysis for AR protein levels in hepatocytes and mHSC isolated from MCD mice (left panel). Images were obtained from separate gels, run under the same experimental conditions, blotted on separate PVDF membranes and processed for detection with the respective antibodies. Densitometric quantification normalized to β-actin levels (right panel). Images are representative of three independent experiments (* p < 0.05).

Besides proliferation, a characteristic of activated hHSC is the production of matrix proteins such as collagen, and over-exuberant matrix over-production results in fibrosis and cirrhosis^21^. The effect of AR on the induction of collagen in hHSC is not known. We analysed the induction of hHSC collagen 1a2 gene expression in the presence of AR. As shown in Figure 5A, AR increased dose-dependently hHSC collagen 1a2 gene expression of 2 to 3 fold in primary hHSC. Similar findings were obtained for ASMA mRNA expression (*data not shown*). The effect is comparable to the effect of the pro-fibrogenic cytokine TGF-β used here as a positive control^22^ (Figure 5A).

To confirm the *in vivo* relevance of these cellular studies, C57/B6 mice were fed for 4 weeks either normal chow or a methionine choline-deficient (MCD) diet, a well-known regimen inducing NASH^23^,^24^. Mice fed a MCD diet had a more abundant hepatic mRNA expression of AR of about 5 times compared to controls (Figure 5B). To discern if the increase in hepatic AR was due to an increase of its expression in hepatocytes (the predominant hepatic cell type) or in HSC, we isolated hepatocytes and HSC (mouse HSC, mHSC) from the livers of control c57/B6 mice and mice fed a MCD diet, and measured AR protein expression by immunoblotting (Figure 5C): while AR protein expression was detected in hepatocytes at similar levels in control and MCD fed mice, it was significantly higher in mHSC and enhanced in mHSC isolated from the NASH model (Figure 5C), confirming mRNA data and highlighting a predominant role of mHSC in NASH-dependent increase in AR expression.

### AR is increased in human patients scored for NASH

**Figure 6:**
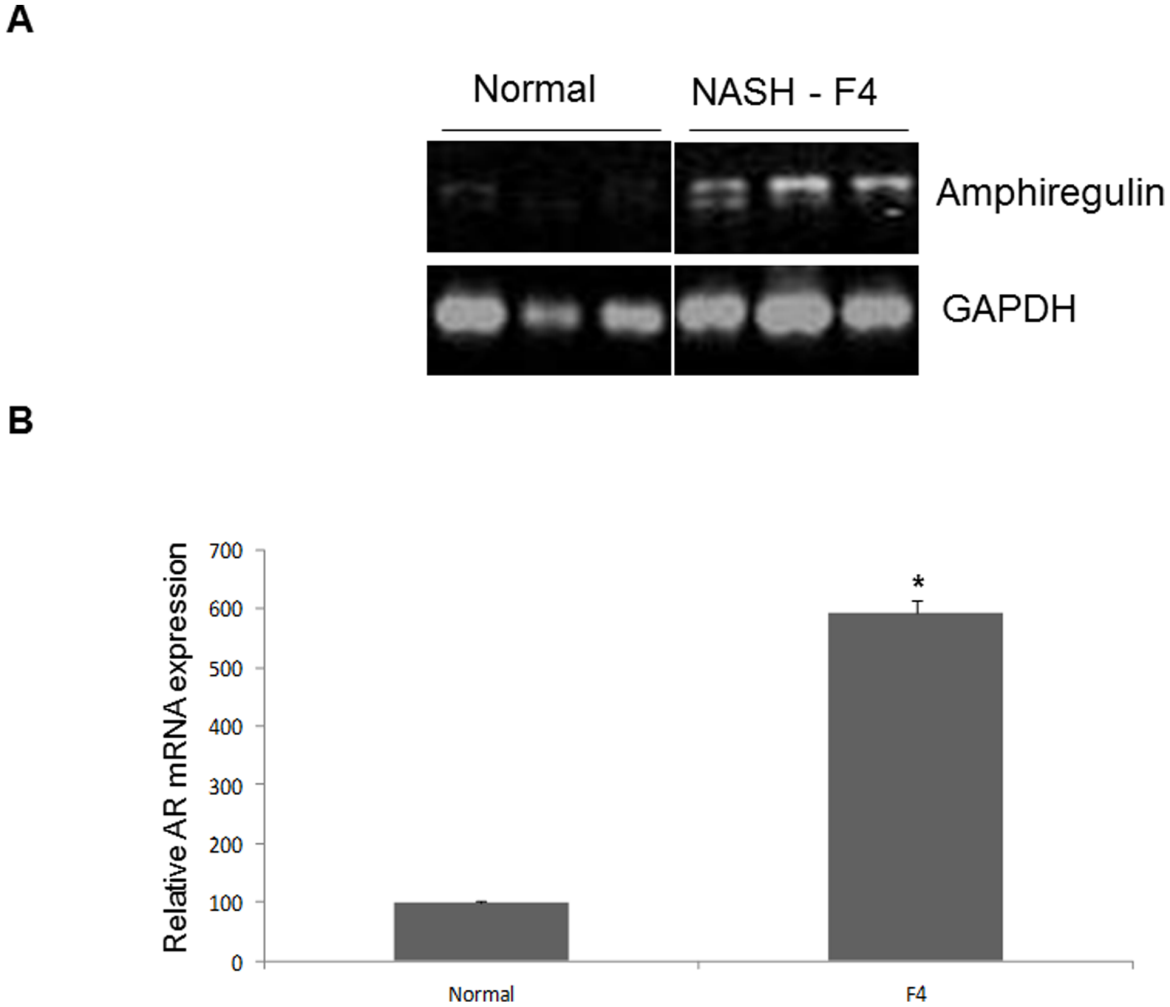
AR mRNA is expressed in patients scored for fibrosis according to both Brunt and NAFLD Activity Score (NAS) of fibrosis severity. (A) Either healthy liver segments of patients undergoing resection of metastases or liver biopsy specimens of patients scored for NASH with fibrosis (grade 4 according to both Brunt and NAS Score of fibrosis severity) were used to assess AR mRNA expression. Control sections express faint AR levels while in NASH patients AR levels are considerably elevated. (B) A comparison of samples shows that relative AR expression is six times higher in NASH livers than in control samples (*p < 0.001).

To study in humans the *in vivo* relevance of these *ex vivo* and animal studies, we analyzed specimens obtained from healthy liver segments of patients undergoing resection of metastases and compared them to samples from patients with histologically proven NASH scored for fibrosis according to both Brunt and NAFLD Activity Score (NAS) of fibrosis severity^25^,^26^. Semi-quantitative RT-PCR analysis of liver RNA from controls and from patients with NASH induced cirrhosis demonstrated a marked upregulation of AR in human livers with NASH compared with controls, where it was only minimally expressed (Figure 6A). Quantification of AR expression in these samples confirmed that AR expression was approximately 6-fold greater in NASH livers compared to controls (Figure 6B).

**Figure 7:**
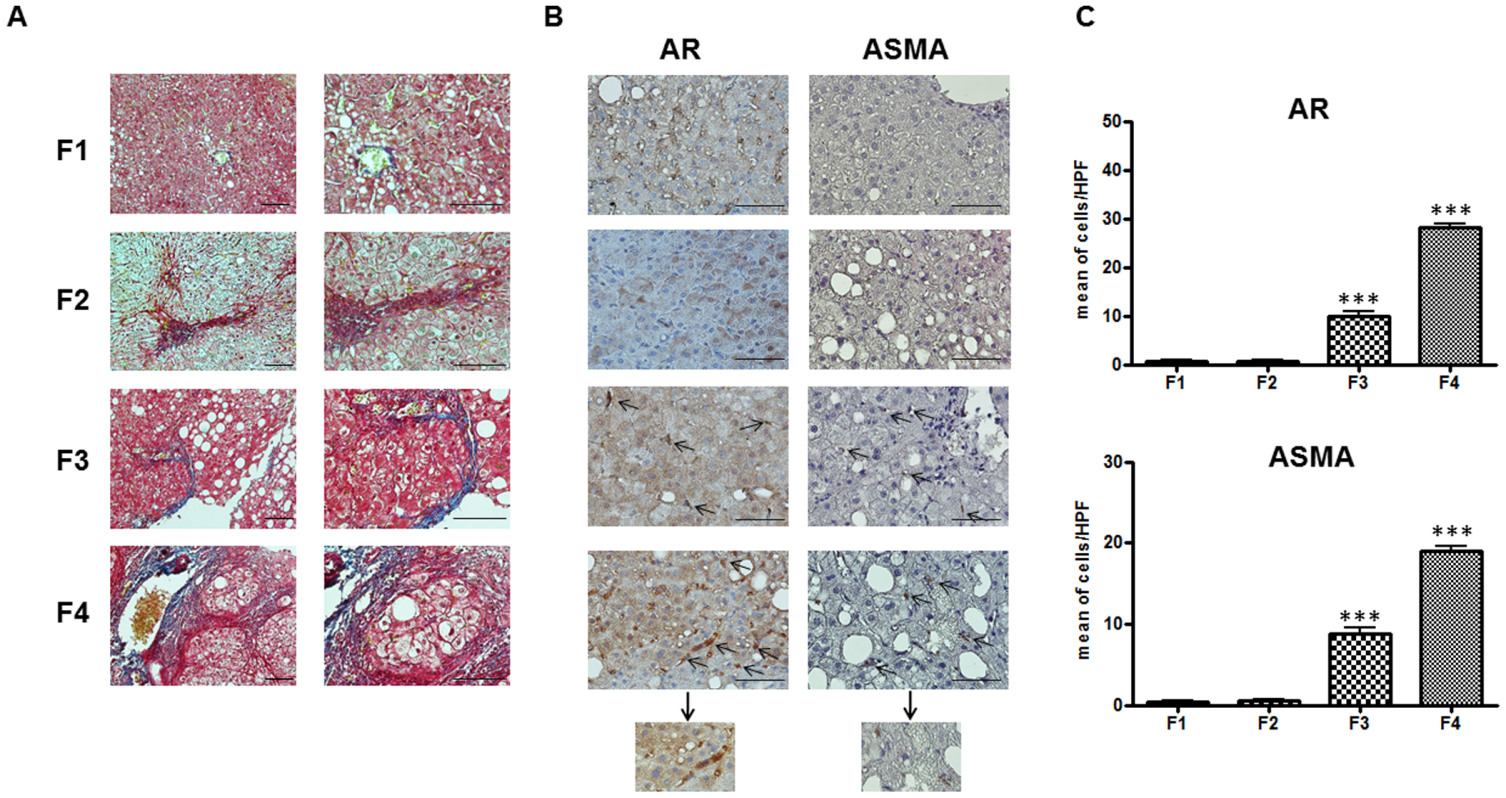
AR and ASMA expression in liver sections in fibrotic patients with increasing NASH severity (F1 to F4), according to both Brunt and NAS Scores; (n = 10). (A) Representative pictures of Masson trichrome staining for collagen (green/blue) in samples with fibrosis F1 to F4. Bar: 100 μm. Magnification 400×. (B) Representative pictures of AR and ASMA immunostainings. Bar: 100 μm. Arrows indicate positive cells. Magnification 400×; highlighted insets were obtained with a magnification 1000×. (C) The quantification values of AR and ASMA immunopositive cells refer to the average number of positive cells for High Power Fields (HFP, 400×). Three independent observers examined the specimens. The means of triplicate counts were used for statistical analyses.

These results were corroborated by immunohistochemical analyses of liver biopsies from patients with increased fibrosis scores (F1, F2, F3 and F4), as visualized by Masson trichrome stainings (Figure 7A). Immunopositivity for AR and for fibrogenic marker ASMA in hHSC was present and abundant only in F3 and F4, with F4 > F3, whereas virtually no positive hHSC were detected in F1 and F2 (Figure 7B and 7C).

## Discussion

**Figure 8:**
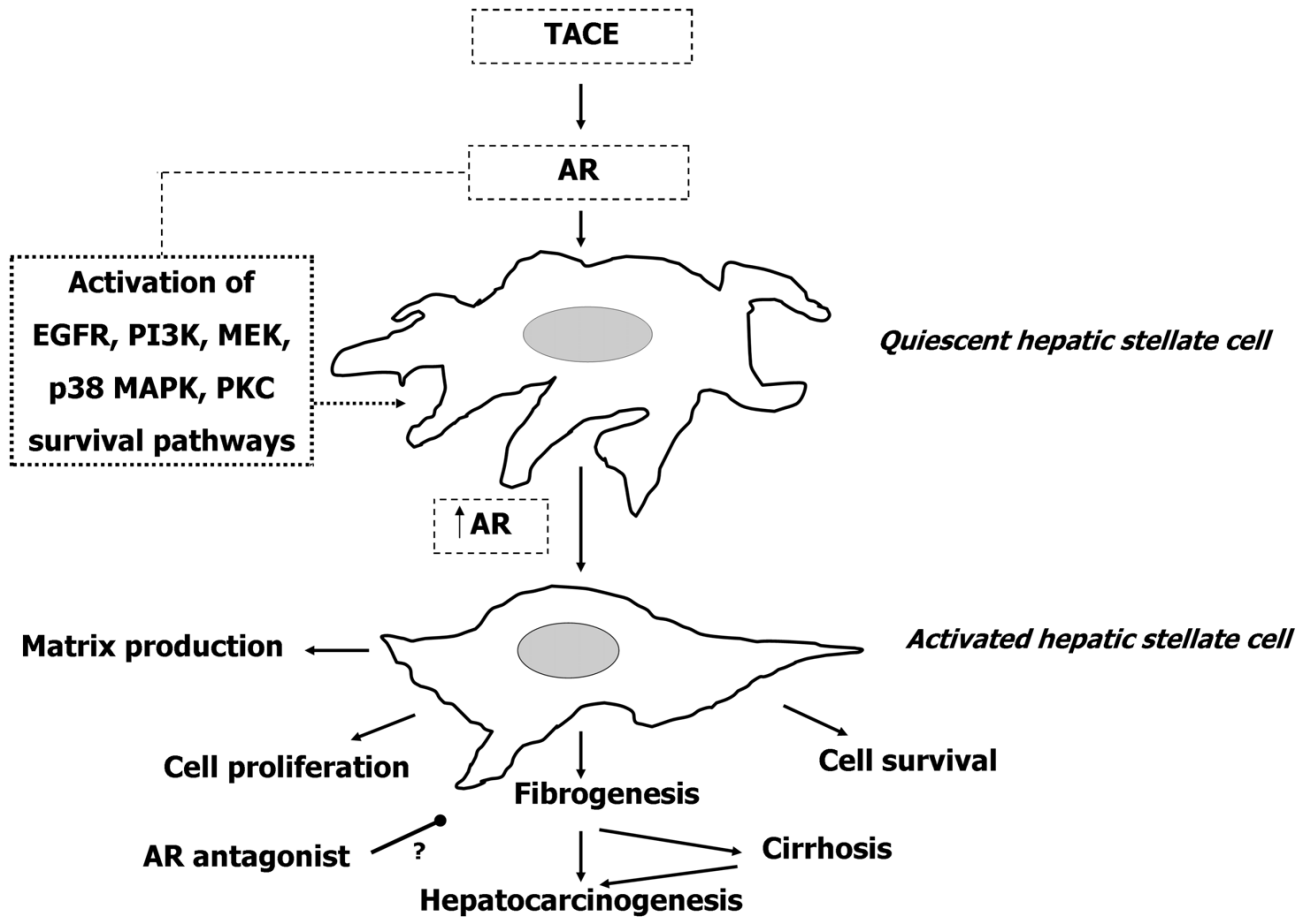
Scheme illustrating the signaling events induced by amphiregulin and influencing hepatic stellate cell dynamics and fate. Continuous lines ending with arrows render positive interactions, while continuous line ending with sphere renders negative interaction. TACE = tumour necrosis factor (TNF) α converting enzyme; AR = amphiregulin.

HSCs have previously been identified as the primary subtype responsible for remodeling liver tissue following injury^27^,^28^. However, the factors that stimulate their proliferation are not fully understood. AR, a protein member of the EGFR-ligand family, is shown here to act both as an hHSC mitogen in primary culture and also to influence the production of hHSC matrix proteins (Figure 8). AR was also shown to be up-regulated in a murine model of NASH and in patients with liver scored for advanced levels of fibrosis and cirrhosis, suggesting that AR may be an important factor in a spectrum of liver diseases ranging from NAFLD trough NASH to cirrhosis. This is fully consistent with the association of AR expression and liver disease that has been previously shown in different animal models of acute liver damage^15^,^16^ and in human samples with cirrhosis and HCC^14^. AR has been widely described as a protein capable to induce mitosis^29^. A link to human diseases was initially reported when AR was found to be increased in patients with lung fibrosis^30^. The role of AR in the liver is under investigation but has been reported to assist in the recovery of hepatic tissues following injury^10^,^12^. This observation is interesting in respect to the liver, which defaults to hepatic fibrogenesis as a means of regenerating injured tissue. Therefore, the question is whether AR is expressed in the restorative HSCs and AR level change upon HSCs activation is relevant.

AR is expressed as a 252 amino acid glycoprotein that exists stably in an uncleaved pro-form^31^,^35^. AR activation has been shown to require cleavage *via* the matrix-metalloproteinase TACE (also known as ADAM17), allowing “shedding” of the ligand from its plasma membrane anchoring and subsequent interaction with cognate receptors^17^. Correspondingly, TACE mRNA levels were observed to increase early in primary hHSC (day 2) and precede the expression of AR (day 4), suggesting its activation from the pro-form (Figure 1A, B). The rise in AR expression mirrors an increase in ASMA during this period, indicating an association between hHSC activation and AR expression. The activation of AR might be expected to induce proliferation in HSC. AR is most commonly identified as a growth factor and acts as a mitogen in a varied range of tissues, including keratinocytes, renal cells, and mammary epithelial cells^31^,^32^,^33^,^34^. Consistently, HSC of AR knock-out mice show an inability to proliferate at the same level as the ones of wild type mice^9^,^10^. As expected, the exogenous addition of AR in the picogram range was sufficient to stimulate proliferation of hHSC. Furthermore, in addition to greater hHSC growth, live cell populations were increased while apoptosis levels did not change significantly (*data not shown*). Thus, AR can act as a survival factor when expressed in liver. The survival pathway by which AR operates has only been partially described. AR has been previously shown to interact with EGFR in various cell types and EGFR expression may in fact constitute a requirement for liver recovery^35^. Data are emerging that AR is expressed at the onset of hepatocyte injury within the liver either by partial hepatectomy or exposure to carbon tetrachloride^10^. The protein is assumed to act through AKT and potentially through one or more mitogen-activated protein (MAP) kinase signaling pathways^19^. However, previous research has limited investigations to only a few potential pathways. In this study, numerous inhibitors have been employed to investigate a broad spectrum of survival pathways including G-coupled proteins, MAP kinases, and mitogens. Each inhibitor was shown to decrease the ability of AR to induce a proliferative response compared to the addition of AR alone. The results agree with previous reports that AR-EGFR complexes are mediated through MAP kinase pathways, and our investigations reveal that specifically the p38 and PI3 kinase pathways were the most affected by inhibitor treatment.

Our work and others have suggested that AR is capable of inducing proliferation in multiple cell types. Additionally, our investigations revealed that collagen 1α, ASMA and positive control TGF-β mRNA are increased upon stimulation of AR in hHSCs (Figure 4 and *data not shown*). Collagen 1α is important for recovery of damaged liver tissues, but its over-production is a clinical hallmark of fibrosis^36^,^37^. One question our investigation sought to answer was whether AR was expressed in a model of liver injury. To understand if AR expression is also associated with liver disease, its expression was investigated initially in mice fed a MCD diet, known to induce fat accumulation in the liver. The model, within its limitations, is able to provoke NASH. Mice fed a MCD diet in fact showed AR up-regulation associated with the presence of NASH specifically in HSC rather than in hepatocytes. These data were corroborated by the evaluation of biopsy specimens obtained from resected human livers scored for increased levels of fibrosis and associated cirrhosis according to both Brunt and NAFLD Activity Score (NAS) of fibrosis severity (scored from 1 to 4; 1 = centrilobular/perisinusoidal; 2 = centrilobular plus periportal; 3 = bridging; 4 = cirrhosis). The mRNA expression of AR in F4 NASH patients was found to be at a level seven times higher respect to that of liver obtained from patients not exhibiting NASH. Consistently, the number of HSC immunopositive for AR in NASH patient liver histological sections was abundant and only detectable in NASH stages F3 and F4. It is known that AR expression is minimal in hepatocytes and other cell types under normal conditions^10^. In agreement with this 1) AR mRNA was detectable but at very low levels in human liver without fibrosis while in NASH F4 livers AR gene expression was much higher; 2) at the protein levels, in NASH F1 and F2, HSC cells positive for AR where very rare or absent, as determined by immunohistochemistry. As livers with grade 3 and 4 scores are very fibrotic, the expression profiles make a strong case that AR is up-regulated in response to damage to the organ and may be important for wound healing. However, an over-expression may contribute to development of fibrosis and onset of cirrhosis.

Fibrosis and progression to cirrhosis have been identified as positive factors in the development of cancer, specifically HCC. Currently, why this relation exists is not known. However, proteins promoting HSC activation and proliferation may act as a contributor to the process. HSCs were recently shown to express WNT and Hedgehog proteins, both of which have been linked to signaling pathways involved in carcinogenesis^38^,^39^. Furthermore, human AR is believed to be a target of WNT as it possesses a binding site for the protein in its promoter^40^. Thus, mitogens expressed in hHSC such as AR may aid in cancer progression. In support of this, AR knock-out animals or knock-downs cell lines have shown a lesser incidence of HCC characteristics^10^,^11^. A future approach would therefore be to determine whether manipulation of AR would be useful as an HCC preventative agent in the treatment of patients with cirrhosis.

In conclusion, AR is implicated in liver regeneration, NAFLD and its fibrogenic form NASH are HCC risk factors, amd hepatic fibrogenesis involves activation of HSC. We show here that AR activates HSC function in mouse and human NASH. With relatively few investigations relating to AR in liver function, the reported results may be of value in therapeutic applications. We present for the first time that AR is expressed in hHSC. Our results have also shown that AR may activate and induce proliferation in hHSC and enhance live cell populations in primary culture. In keeping with what is known about stellate cells in liver, their increased proliferation is thought to be aided by AR during liver wound healing, likely producing a protective layer of extra-cellular matrix (ECM) that supports repopulating hepatocytes. The data compiled here supports the hypothesis, as collagen and ASMA production is concurrent with AR-induced proliferation of hHSC. The observation is complemented in both a mouse model of NASH and patient biopsies with NASH. The human data lends added importance to this study as we suggest that AR expression may be a component of the liver's survival response to NAFLD. However, overproduction of AR may lead to increased levels of fibrosis and lead to cirrhosis and potentially HCC. Thus, regulation of AR may be used to control the severity of the disease, using a regime that will promote liver recovery but control overstimulation and the potential formation of fibrosis.

## Methods

### Animals

Adult C57/BL6 animals (Charles River Laboratories, UK) 8 to 10 weeks old, were used. All animal studies were approved by the University Hospitals of Leuven, Belgium, and all the procedures described have been carried out in accordance with EU Directive 2010/63/EU for animal experiments. The methionine-choline deficient (MCD) model is an established dietary model of murine NASH^23^. After 4 weeks of MCD or a control diet, mice were sacrificed by asphyxiation with CO_2_ and cervical dislocation.

### Human Biopsies and Histological Evaluations

Liver specimens were obtained from patients undergoing clinically indicated hepatic biopsies, with the approval of the University of Palermo (Department of Pathology), patient consent and in accordance with the Code of Ethics of the World Medical Association (Declaration of Helsinki) for experiments involving humans. Liver biopsy specimens were scored for the presence of NAFLD according to the Brunt and NAFLD Activity Score (NAS) of fibrosis severity^26^,^27^, by a blinded expert liver pathologist. Liver tissue obtained from biopsy specimens were flash frozen in liquid nitrogen and stored at minus 80°C or, alternatively, formalin-fixed paraffin embedded until further use. 10 cases were selected for each stage of fibrosis score/severity (F1 to F4). From the paraffin blocks, sections of human liver biopsies with a thickness of 4–5 mm were obtained using a cutting microtome. After the sections were stained by Masson trichrome staining for histological evaluation as previously described^43^.

### Immunohistochemistry

Immunostainings were performed by iVIEW DAB Detection Kit for Ventana BenchMark XT automated slide stainer on human biopsies^43^. Primary antibodies for AR (H-155, Santa Cruz Biotechnology) and ASMA (ACTA-2, SIGMA) were diluted 1:100. Positive and negative controls were run concurrently. For assessment of AR and ASMA cell immunopositivity, the value expressed refers to the average number of positive cells for High Power Fields (HPF, 400×). Three independent observers examined the specimens. All the observations were made at a magnification 400× and the means of triplicate counts were used for statistical analyses.

### Isolation and Culture of Human Primary Hepatic Stellate Cells

hHSC were obtained, with appropriate local Ethical approval and patient consent, from healthy liver segments of patients undergoing metastases resection. hHSCs were isolated by collagenase (type IV) perfusion, followed by pronase treatment, as previously described^21^,^41^. Cell identity was confirmed by autofluorescence, and expression of α-smooth muscle actin (ASMA), an accepted HSC marker. Experiments were performed with primary hHSCs in culture as previously described^42^.

### Isolation and Culture of Mouse Primary Hepatic Stellate Cells and Hepatocytes

Mouse HSCs (mHSCs) were isolated from the livers of wild type and MCD male mice by in situ perfusion using collagenase and pronase^41^,^44^. The viability and purity of HSC preparations were consistently found to be >95% as accessed via trypan blue (Gibco-BRL, Grand Island, NY, USA) exclusion and autofluorescence, respectively. Hepatocytes were isolated from wild-type and MCD male mice, purified by Percoll gradient centrifugation and cultured as previously described^41^,^44^.

### Immunocytochemistry

hHSC were harvested at days 0, 2, 4, and 10. Subsequently, the cells were fixed in 50% ethanol/acetic acid and cooled for 10 min at −20°C. Fixing buffer was then removed and the cells washed with PBS and processed for antigen unmasking using 10 μM sodium citrate and 0.2% Triton X. After blocking with 10% goat serum (Vector Labs, s-1000) at room temperature for 20 min, the protein of interest was labeled by incubating the cells overnight at 4°C with a mouse monoclonal primary antibody (ASMA, sc-32251, AR, sc-74501, Santa Cruz Biotechnology) at a 1:400 dilution. The primary antibody binding was later detected using the appropriate anti-mouse secondary antibody (1:10,000 dilution) conjugated with FITC. DAPI was used in the mounting medium to stain the nuclei and samples examined by confocal microscopy.

### Drugs

EGFR inhibitor PD 153035, pertussis toxin (PT), wortmannin (WT), PD98059 (PD), SB202190 (SB), and Ro-32-0432 (RO) were obtained from Sigma and were used on hHSC at concentration previously described^20^.

### Cell Proliferation Assay

Basal and induced hHSC proliferation was determined using a colorimetric assay^45^. Optical densities were read from 96-well plates with an Emax precision microplate reader.

### Apoptosis Assay

Equal numbers of hHSC were plated on 6 mm petri dishes with or without AR. At harvest, apoptotic activity was assessed with the Vybrant (annexin V) apoptosis assay kit 2 (Molecular Probes, InVitrogen). Flow cytometric analysis was performed using a Becton-Dickinson flow cytometer.

### RT-PCR

RNA was isolated from activated hHSCs using Trizol reagent (Invitrogen) and DNA generated with Superscript III one-step RT-PCR with platinum Taq kit (Invitrogen, cat.10928034). Relative quantification of the mRNAs was performed on a Rotorgene RG-3000 instrument (Corbett Research). Collagen 1-α2, TGF-β1, ASMA, AR and GAPDH primers were custom made (InVitrogen) while classic II 18S internal standard kit was from Ambion (Applied Biosystems, UK). Primers sequences, product sizes and Tm were as follows: **Collagen 1α2:** sense 5′-ATA TTG CAC CTT TGG ACA TC 3′, antisense 5′-TGC TCT GAT CAA TCC TTC TT-3′ (236 bp, 55°C); **TGF-β1:** sense 5′-AAC CCA CAA CGA AAT CTA TG and antisense 5′-GTG CTG CTC CAC TTT TAA CT-3′ (157 bp, 55°C).

**AR:** sense 5′-GAC ACC TAC TCC GGG AAA GCG TG-3′ and antisense 5′-AGC CAG GTA TTT GTG GTT CG-3′ (196 bp, 55°C); **TACE:** sense 5′-GAA GTG CCA GGA GGC GAT TA-3′ and antisense 5′-CGG GCA CTC ACT GCT ATT ACC-3′ (73 bp, 55°C); **GAPDH**: sense 5′-ACA GTC CAT GCC ATC ACT GCC-3′ and antisense 5′-GCC TGC TTC ACC ACC TTC TTG-3′ (266 bp, 55°C). To monitor specificity, final PCR products were analyzed both by melting curves and agarose gel (1%) electrophoresis. The amount of transcript was calculated and expressed as the difference relative to the control gene GAPDH (2^−ΔΔCt^, where ΔCt represents the difference in threshold cycles between the target and control genes).

### Western Blotting

Total caspase-3 was evaluated by western blot analysis, as previously described^46^. Briefly, samples were homogenized with RIPA buffer (NaCl, Tris-HCL, 1% sodium deoxycholate, 0.1% SDS, and 1% NP-40, Sigma) plus protease cocktail (Roche). Samples (≥20 μg protein) were then loaded onto a 10% bis-tris polyacrylamide gel (Invitrogen, NP301). Western blots were performed using as primary antibodies caspase-8 (Cell Signaling #9746), β-actin (Santa Cruz Biotechnology, Santa Cruz, Ca, SC-1616), ADAM17/TACE (Abcam, ab77820) and amphiregulin (Thermo Scientific, PA5-27298), pEGFR (Y1068) (Abcam, ab5644), pAKT (Ser473) (Cell Signaling #9271), pERK1/2 (Thr202/Tyr204) (Cell Signaling #9101) and p-p38 (Thr180/Tyr182) (Cell Signaling #9211) at a dilution of 0.5 μg/ml. Horseradish peroxidase-conjugated secondary antibodies (SantaCruz Biotechnology, SC-2031) were used to detect primary antibodies at a 1:10,000 dilution ratio. Signals were detected by an ECL kit (Amersham Pharmacia Biosciences, Piscataway, NJ). Equal loading (20 μg) was verified by comparing expression levels of β-actin.

### TNF-α converting enzyme (TACE) activity

TACE activity was assessed in cultured hHSC using the commercial fluorimetric SensoLyte® 520 TACE (α-Secretase) Activity Assay Kit (Anaspec, CA, USA), according to manufacturer's instructions.

### Statistical Analysis

All data are expressed as mean (±SEM) of at least 3 separate experiments. Statistical analyses were performed using the Mann-Whitney test. A p value was considered statistically significant if p < 0.05.

## Author Contributions

Study concept, design and supervision: J.A.O.; Acquisition of data: C.M., B.S., J.S., A.M., M.M., F.R. and V.P.; Analysis and interpretation of data: F.C., V.P., C.M., B.S., J.S., A.M., M.M., M.V. and J.A.O.; Drafting of the manuscript: G.M., M.V. and J.A.O.; Statistical analysis: G.M.; Obtained funding: M.V. and J.A.O.; Technical support: D.C., C.S. and T.R.
